# The Severity of Fecal Problems Is Negatively Associated With Quality of Life in a Dutch Population Without Bowel Function Comorbidities

**DOI:** 10.1097/DCR.0000000000003048

**Published:** 2023-11-02

**Authors:** Maaike B.C. ten Hoor, Monika Trzpis, Paul M.A. Broens

**Affiliations:** 1 Department of Surgery, Anorectal Physiology Laboratory, University of Groningen, University Medical Center Groningen, Groningen, The Netherlands; 2 Division of Pediatric Surgery, Department of Surgery, University of Groningen, University Medical Center Groningen, Groningen, The Netherlands

**Keywords:** Constipation, Fecal incontinence, Fecal problems, Quality of life

## Abstract

**BACKGROUND:**

Constipation and fecal incontinence negatively influence quality of life. The association between the severity of fecal problems and quality of life has not been investigated in the general population without bowel function comorbidities.

**OBJECTIVE:**

To investigate the association between the severity of constipation and fecal incontinence and quality of life in patients without comorbidities influencing bowel function.

**DESIGN:**

A population-based, cross-sectional study.

**SETTINGS:**

The study involved 3668 Dutch study participants.

**PATIENTS:**

A survey company conducted a population-wide study of the general Dutch population. Altogether, 5000 Dutch citizens completed the Groningen Defecation and Fecal Continence and Short Form-36 questionnaires. The data on 3668 respondents without comorbidities that could influence bowel function were included for analysis (study group).

**MAIN OUTCOME MEASURES:**

The severity of constipation (Agachan score) and fecal incontinence (Wexner score) in relation to the quality-of-life scores.

**RESULTS:**

In the study group (n = 3668), 487 had constipation (13.3%), 116 had fecal incontinence (3.2%), and 64 had 2 coexisting fecal problems (1.7%). In the multivariable analysis, all quality-of-life domains were negatively associated with the severity of constipation and fecal incontinence. The associations between the severity of constipation and quality of life were stronger (highest: ß = –2.413; 95% CI, –2.681 to –2.145; *p* < 0.001) than those of fecal incontinence (highest: ß = –1.280; 95% CI, –1.681 to –.880; *p* < 0.001). We also found that a longer duration of bowel complaints coincided with higher severity scores, especially for constipation. Respondents mostly rated their defecation health as positive, regardless of the severity of their fecal problems.

**LIMITATIONS:**

Cross-sectional design.

**CONCLUSIONS:**

The severity of constipation and fecal incontinence is significantly associated with reduced quality of life, with the severity of constipation having stronger associations than fecal incontinence. Given respondents’ unawareness of their fecal problems and the progressive character, timely intervention is advocated. See **Video Abstract**.

**LA GRAVEDAD DE LOS PROBLEMAS FECALES SE ASOCIA NEGATIVAMENTE CON LA CALIDAD DE VIDA EN UNA POBLACIÓN HOLANDESA SIN COMORBILIDADES DE LA FUNCIÓN INTESTINAL:**

**ANTECEDENTES:**

El estreñimiento y la incontinencia fecal influyen negativamente en la calidad de vida. La asociación entre la gravedad de los problemas fecales y la calidad de vida no se ha investigado en la población general sin comorbilidades de la función intestinal.

**OBJETIVO:**

Investigar la asociación entre la gravedad del estreñimiento y la incontinencia fecal y la calidad de vida en la población holandesa general sin comorbilidades que influyan en la función intestinal.

**DISEÑO:**

Estudio transversal de base poblacional.

**ENTORNO CLINICO:**

El estudio involucró a 3668 participantes holandeses.

**PACIENTES:**

Una empresa de encuestas realizó un estudio poblacional de la población holandesa en general. En total, 5.000 ciudadanos holandeses completaron los cuestionarios Groningen Defecation and Fecal Continence y Short-Form 36. Se incluyeron para el análisis los datos de 3668 encuestados sin comorbilidades que pudieran influir en la función intestinal (grupo de estudio).

**PRINCIPALES MEDIDAS DE RESULTADO:**

La gravedad del estreñimiento (puntuación de Agachan) y la incontinencia fecal (puntuación de Wexner) en relación con las puntuaciones de calidad de vida.

**RESULTADOS:**

En el grupo de estudio (n = 3668), 487 tenían estreñimiento (13,3%), 116 tenían incontinencia fecal (3,2%) y 64 tenían 2 problemas fecales coexistentes (1,7%). En el análisis multivariable, todos los dominios de calidad de vida se asociaron negativamente con la gravedad del estreñimiento y la incontinencia fecal. Las asociaciones entre la gravedad del estreñimiento y la calidad de vida fueron más fuertes (más alta: ß = –2,413; IC del 95 %, –2,681 a –2,145; p < 0,001) que las de la incontinencia fecal (más alta: ß = –1,280; 95 IC %: –1,681 a –0,880; p < 0,001). También encontramos que una mayor duración de las molestias intestinales coincidió con puntuaciones de gravedad más altas, especialmente para el estreñimiento. La mayoría de los encuestados calificaron su salud en la defecación como positiva, independientemente de la gravedad de sus problemas fecales.

**LIMITACIONES:**

Diseño transversal.

**CONCLUSIONES:**

La gravedad del estreñimiento y la incontinencia fecal se asocia significativamente con una calidad de vida reducida; la gravedad del estreñimiento tiene asociaciones más fuertes que la incontinencia fecal. Dado el desconocimiento de los encuestados sobre sus problemas fecales y el carácter progresivo, se recomienda una intervención oportuna. *(Traducción— Dr. Francisco M. Abarca-Rendon*)

In a 2017 study of the general Dutch population, 24.5% of individuals reported constipation, 7.9% reported fecal incontinence (FI), and 3.5% reported a combination of both.^1^ The prevalence of both constipation and FI may be underestimated because patients tend to underrecognize their fecal problems. For example, 48.7% of the respondents with constipation and 35.0% with FI rated their bowel habits as “good” or “really good.”^1^ Underestimating fecal problems could also result from individuals’ reserve to disclose these problems to health care professionals.^1–3^ Time for adequate anamnesis is often limited, which makes it difficult for physicians to determine which patients have fecal problems. Hence, multiple factors lead to underestimating fecal problems.^4^ Although a few studies have reported an association between the presence of constipation and/or FI and reduced quality of life (QoL),^3,5–7^ little is known about the association between the severity of constipation or FI and QoL. However, these studies show ambiguous outcomes and are restricted to patient populations.^6,7^ A few studies have documented fecal problems in the general population. Nevertheless, only a limited number of publications are dedicated to constipation, FI, and their coexistence in relation to QoL and its different domains of people struggling with such problems.^1,8^ Respondents in the general population without bowel function comorbidities are not eager to discuss their fecal problems, especially if such problems are mild.^1^ However, mild forms of constipation can lead to chronic constipation if left untreated, eventually leading to FI.^1,2^

It seems plausible that less severe forms of fecal problems are less invalidating and will have a less negative effect on QoL than the more severe forms. Soiling, for example, would reduce QoL less than complete FI, in which case someone might avoid public places for fear of fecal spillage. We hypothesized that the more severe the fecal problems are, the lower QoL will be. Our primary aim was to investigate the possible association between the severity of constipation or FI and QoL in the Dutch population without bowel function comorbidities (study group).

## PATIENTS AND METHODS

### Study Design and Participants

This prospective, cross-sectional, descriptive study was performed between September 2021 and July 2022 at the Anorectal Physiology Laboratory of the Department of Surgery of University Medical Center Groningen in The Netherlands. The Medical Ethics Review Board of the University Medical Center Groningen approved this study (M.23.315004). At our request, Dynata (Rotterdam), an international survey company, collected data on 5000 respondents from the general Dutch population. The distribution of the sample across age, sex, residency, and educational level was normal and representative of the general Dutch population. The database we obtained from the company was anonymous. It was not possible for respondents to withdraw consent because there was no direct communication between us and the respondents. This population-wide survey included the validated Defecation and Fecal Continence (DeFeC) and the Short Form-36 (SF-36) questionnaires. The DeFeC questionnaire contains a scoring system that determines the severity of constipation and FI.^9^ It starts by determining the respondents’ self-perception of defecation health. The subsequent questions address anorectal functioning, associative diseases, and causative factors.^9^ The SF-36 questionnaire measures perception of health on 8 multi-item dimensions: functional status (physical and social functioning, role limitations due to both physical and emotional problems), well-being (mental health, vitality), and overall evaluation of health (general health perception, health change).^10^

We excluded respondents who had comorbidities known to influence bowel function, thus preventing QoL scores from being affected by coexisting bowel problems. Diseases we excluded were IBD, irritable bowel syndrome, prolapse of the rectum, diabetes mellitus, brain hemorrhage or stroke, other neurological disorders such as multiple sclerosis and paraplegia, slow transit constipation, congenital anorectal malformation, Hirschsprung’s disease, sacrococcygeal syndrome, and spina bifida. We also excluded respondents who had undergone surgery known to influence bowel function, such as surgery of the bowel, pelvic floor, and anal canal/sphincter.

The remaining respondents formed the study group, which we subsequently divided into 4 subgroups according to the Rome IV criteria: constipation, FI, coexisting constipation and FI, and no fecal problems subgroups. We also analyzed the duration of bowel complaints (ie, having difficulty losing fecal contents) and self-estimation of defecation health in different severity subgroups. In the constipation group, we distinguished between respondents whose severity scores ranged between 0 (no constipation), 1 to 15 (between no constipation and the clinically relevant threshold),^11^ and 15 or higher (clinically significant constipation). We did the same for FI, in which the subgroups comprised respondents with a severity score of 0 (no FI), between 1 and 8 (between no FI and the threshold), and between 9 and 20 (clinically significant FI).^12^

### Outcome Measures

The primary outcomes were the severity of constipation (Agachan score), the severity of FI (Wexner score), and QoL scores. The questions concerning FI and constipation-related symptoms in the DeFeC questionnaire enabled us to assess the Agachan and Wexner scores and the prevalence of FI and constipation based on the Rome IV criteria.^9^ The Agachan constipation score comprises 8 items: frequency of bowel movements, painful evacuation efforts, feeling incomplete evacuation, abdominal pain, time in minutes spent in the lavatory per attempt, type of assistance, unsuccessful attempts at evacuation per 24 hours, and duration of constipation in years. Items are graded on a 5-point Likert scale except for the item “type of assistance.”^11^ Scores range from 0 (no constipation) to 30 (severe constipation). The Wexner incontinence score includes 5 items: incontinence of flatus, incontinence of liquid, incontinence of solid, wearing an incontinence pad, and lifestyle requirements.^13,14^ Scores range from 0 (normal) to 20 (complete FI). We used the SF-36 questionnaire to assess QoL. It comprises 36 questions in 8 domains: physical functioning, social functioning, role limitations due to physical problems, role limitations due to emotional problems, mental health, vitality, pain, and general health perception.^15^ This questionnaire also calculates a Likert scale, with scores ranging from 0 to 100, with a higher score indicating better QoL.

The secondary outcome was a comparison of the magnitudes of the associations between the severity of fecal problems and QoL. Following multivariable linear regression, we compared the unstandardized regression coefficients of each QoL domain with the severity of constipation or FI.

### Statistical Analysis

Descriptive statistics were used to describe the characteristics of the study group. The Q-Q plot tested the normal distribution of continuous variables. Given the large sample size, we performed tests for normal distributions.^16^ Continuous variables were therefore presented as means with SDs and compared using the *t* test. Correlations were calculated using Pearson’s correlation. The categorical variables were presented as percentages. Associations of categorical variables were made using the χ^2^ test. Multivariable linear regression analysis determined the unstandardized regression coefficient corresponding to 95% CI regarding the severity of constipation and FI and the QoL domain scores. We chose unstandardized regression coefficients because, in this way, the relationship between the severity of fecal problems and QoL is more visible and more accessible. The unstandardized regression coefficient measures the association between the severity of constipation and the severity of FI and QoL. Statistical significance was determined at a probability of <0.05. All the statistical tests were using SPSS statistics, version 23.0 (IBM Corporation, Armonk, NY). The figures were generated using GraphPad Prism, version 9.4.1 (Graphpad Software, San Diego, CA).

## RESULTS

### Characteristics of the Study Group

From our database of 5000 respondents, we excluded 9 because of illogical answers. Another 1323 were excluded because they had comorbidities related to bowel function. The remaining 3668 respondents constituted the study population and comprised 1871 (51.0%) women and 1797 (49.0%) men. The mean age was 47.4 (SD ±16.9) years and the mean BMI was 26.0 (SD ±4.9). In this study population, 487 (13.3%) respondents had constipation, 116 (3.2%) had FI, 64 (1.7%) had both fecal problems, and 3001 (81.8%) had neither constipation nor FI (Table 1). In the constipation group, the mean constipation severity score was 6.9 (SD ±4.0) and the mean FI severity score was 2.1 (SD ±1.8; Table 1). In the FI group, the mean constipation severity score was 4.8 (SD ±3.5) and the mean FI severity score was 6.9 (SD ±3.3; Table 1).

**TABLE 1.** The severity of constipation and fecal incontinence in relation to self-perception and quality of life

*Study population characteristics*	*Total*	*Constipation*	*Fecal incontinence*	*Coexisting constipation and fecal incontinence*	*No fecal problems*
No. of participants, n (%)	3668 (100)	487 (13.3)	116 (3.2)	64 (1.7)	3001 (81.8)
Sex, n (%)					
Male	1797 (49.0)	175 (35.9)	56 (48.3)	31 (48.4)	1535 (51.1)
Female	1871 (51.0)	312 (64.0)	60 (51.7)	33 (51.6)	1466 (48.9)
Age, y, mean (SD)	47.4 (16.9)	43.6 (17.1)	44.09 (18.0)	41.8 (16.9)	48.2 (16.7)
BMI, mean (SD)	26.0 (4.9)	25.2 (5.0)	26.9 (6.0)	25.9 (5.6)	26.0 (4.9)
Severity of constipation and fecal incontinence scores, mean (SD)					
Agachan score	3.0 (3.1)	6.9 (4.0)	4.8 (3.5)	9.0 (3.7)	2.2 (2.2)
Wexner score	2.0 (2.1)	2.1 (1.8)	6.9 (3.3)	7.8 (3.0)	1.7 (1.6)
Self-perception regarding fecal problems, n (%)					
Very good	1076 (29.3)	46 (9.4)	18 (15.5)	4 (6.3)	1008 (33.6)
Good	1786 (48.7)	191 (39.2)	48 (41.4)	21 (32.8)	1526 (50.8)
Reasonable	677 (18.5)	183 (37.6)	41 (35.3)	29 (45.3)	424 (14.1)
Poor	119 (3.2)	63 (12.9)	7 (6.0)	9 (14.0)	40 (1.3)
Very poor	10 (0.3)	4 (0.8)	2 (1.7)	1 (1.6)	3 (0.1)
Quality of life scores per domain, mean (SD)					
Physical functioning	86.3 (19.9)	81.9 (23.1)	78.4 (25.4)	68.5 (27.9)	87.7 (18.5)
Social functioning	81.1 (22.5)	73.4 (25.2)	70.8 (26.3)	60.4 (21.3)	83.1 (21.3)
Role limitations (physical)	76.8 (26.3)	68.8 (29.6)	67.8 (28.8)	48.2 (24.2)	79.1 (25.0)
Role limitations (emotional)	79.1 (26.2)	71.6 (28.9)	68.2 (30.7)	51.4 (24.2)	81.3 (24.9)
Mental health	72.7 (18.5)	66.9 (20.2)	64.9 (21.4)	54.8 (17.7)	74.4 (17.7)
Vitality	61.4 (19.1)	54.7 (19.6)	54.0 (20.5)	49.9 (17.6)	63.0 (18.7)
Pain	78.5 (21.2)	72.2 (24.2)	71.8 (21.0)	59.2 (22.3)	80.2 (20.3)
General health perception	65.9 (18.9)	60.6 (20.4)	57.3 (20.1)	50.0 (19.4)	67.5 (18.2)

In constipated respondents, the lowest mean QoL score was observed for vitality at 54.7 (SD ±19.6) and for general health perception at 60.6 (SD ±20.4), and the highest mean QoL score was observed for physical functioning at 81.9 (SD ±23.1) (Table 1). In the FI group, the lowest mean QoL score was observed for vitality at 54.0 (SD ±20.5) and for general health perception at 57.3 (SD ±20.1), whereas the highest mean QoL score was observed for physical functioning at 78.4 (SD ±25.4) (Table 1). Overall, the lowest QoL scores were observed in the group in which constipation coexisted with FI, and the QoL scores of respondents having FI were slightly lower than those having constipation (Table 1). Of the constipated respondents, 86.2% rated their defecation health as very good, good, or reasonable, and 13.7% rated it as poor or very poor. Surprisingly, in the FI group, 92.2% described their defecation health as very good, good, or reasonable, and only 7.7% rated it as poor or very poor. In the group with coexisting constipation and FI, 84.4% rated their defecation health as good, reasonable, or very good, and 15.7% rated it as poor or very poor.

### The Relationship Between the Severity of Constipation and FI and QoL

**FIGURE 1.**
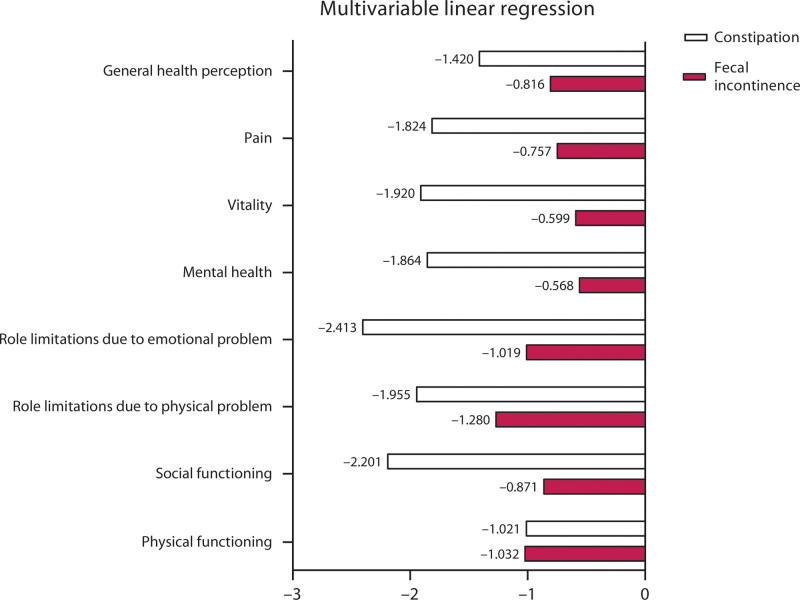
The unstandardized regression coefficients of quality of life domains (representing the magnitudes of the associations) and the severity of constipation and fecal incontinence.

We corrected the outcome for constipation severity when investigating the association between FI severity and the QoL using multivariable linear regression analysis because 1.7% of the respondents had both FI and constipation. Similarly, we corrected for FI severity when investigating the association between constipation severity and QoL. We also corrected the level of education and place of residence because this might influence the QoL of the respondents. The rates of defecation problems per category of the habitat of living and level of education are shown in Table 2. Overall, the association between the severity of constipation and QoL had higher unstandardized regression coefficients than the association between the severity of FI and QoL. This was observed for all QoL domains except physical functioning. Considering the severity of constipation, the unstandardized regression coefficient was highest for role limitations due to emotional problems (ß = –2.413; 95% CI, –2.681 to –2.145; *p* ≤ 0.001) and social functioning (ß = –2.201; 95% CI, –2.430 to –1.972; *p* ≤ 0.001; Fig. 1; see Supplemental Table 1 at http://links.lww.com/DCR/C260). Considering the severity of FI, the regression coefficient was highest for role limitations due to physical problems (ß = –1.280; 95% CI, –1.681 to –.880; *p* ≤ 0.001; Fig. 1; see Supplemental Table 1 at http://links.lww.com/DCR/C260). The unstandardized regression coefficient entails that when the severity score increases by 1 point, the QoL score for that particular domain lowers with the quantity of the unstandardized regression coefficient.

**TABLE 2.** Habitat of living and level of education in relation to the rates of defecation problems

*Sociodemographic characteristics*	*Total*	*Constipation*	*Fecal incontinence*	*Coexisting constipation and fecal incontinence*	*No fecal problems*
No. of participants, n (%)	3668 (100.0)	487 (13.3)	116 (3.2)	64 (1.7)	3001 (81.8)
Place of residence, n (%)					
Village	1200 (100.0)	156 (13.0)	31 (2.6)	15 (1.3)	998 (83.2)
City <25,000 citizens	402 (100.0)	54 (13.4)	18 (4.5)	8 (2.0)	322 (80.1)
City 25,000–50,000 citizens	600 (100.0)	73 (12.2)	25 (4.2)	13 (2.3)	489 (81.5)
City 50,000–100,000 citizens	573 (100.0)	80 (14.0)	19 (3.3)	17 (3.0)	457 (79.8)
City >100,000 citizens	893 (100.0)	124 (13.9)	23 (2.6)	11 (1.2)	735 (82.3)
Educational level, n (%)					
Low	603 (100.0)	78 (12.9)	27 (4.5)	3 (0.5)	495 (82.1)
Middle	1507 (100.0)	200 (13.3)	53 (3.5)	30 (2.0)	1224 (81.2)
High	1558 (100.0)	209 (13.4)	36 (2.3)	31 (2.0)	1282 (82.3)

**FIGURE 2.**
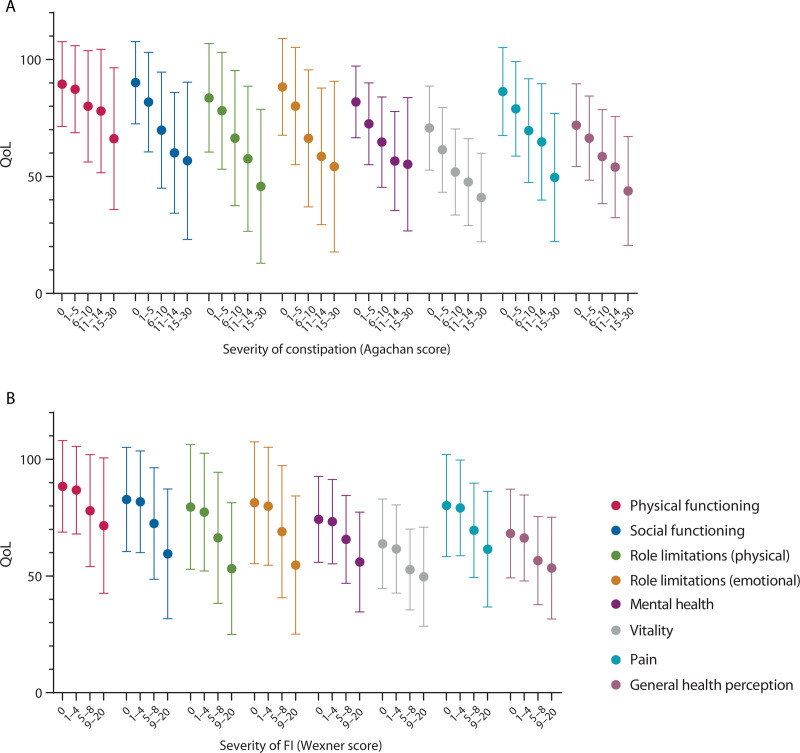
QoL scores and the severity of constipation (A) and FI (B). FI = fecal incontinence; QoL = quality of life.

Furthermore, we found that this observation applied not only to clinically relevant constipation (ie, a constipation severity score above 9) but also to patients with scores between 1 and 6. They also had lower QoL scores than respondents with a constipation severity score of 0 (ie, no constipation; Fig. 2). By contrast, FI respondents with Wexner scores of 0 had QoL scores on most domains comparable to respondents with mild forms of FI (ie, with scores between 1 and 4).

### The Duration of Bowel Complaints and the Severity of Constipation and FI

**FIGURE 3.**
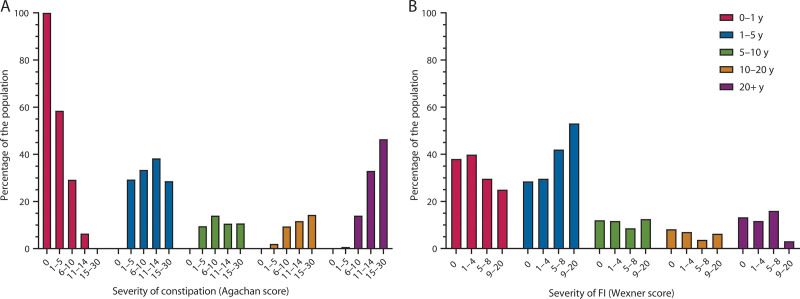
The duration of bowel complaints (ie, problems with losing stools) and the severity of constipation (A) and FI (B). FI = fecal incontinence.

We also found that respondents with the mildest constipation symptoms (ie, Agachan scores between 1 and 5) reported the shortest duration of problems with losing stools, namely 0 to 1 year (Fig. 3). By contrast, respondents with the most severe constipation symptoms (ie, Agachan scores between 15 and 30) reported the longest duration of complaints, namely >20 years. This relationship between severity and duration of symptoms was not observed in respondents with FI. Across all FI severity ranges, the number of respondents who had bowel complaints for a brief time, namely 0 to 1 year or 1 to 5 years, was the highest.

### The Self-Perception of Defecation Health and Severity of Constipation and FI

**FIGURE 4.**
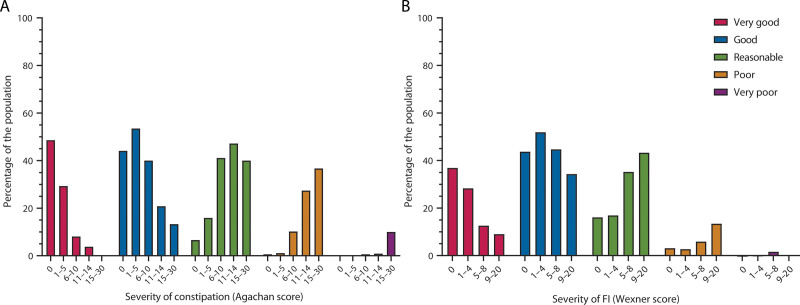
The self-perception of defecation health and the severity of constipation (A) and FI (B). FI = fecal incontinence.

Finally, we found that respondents who rated their defecation health as good or very good decreased with increasing severity of constipation (Fig. 4). Interestingly, in respondents with the most severe constipation (Agachan scores between 15 and 30), 53.3% described their defecation health as good or reasonable (Fig. 4). In FI respondents with the highest severity (Wexner scores between 9 and 20), 86.6% described their defecation health as very good, good, or reasonable. QoL is influenced by many factors. In our study, we found that both the respondents’ environment and educational level are associated with QoL (data not shown). In Table 3, we analyzed self-perceived defecation health per category of habitat of living and level of education.

**TABLE 3.** The associations between self-perceived defecation health, habitat of living, and level of education

*Sociodemographic characteristics*	*Self-perceived defecation health*					
	*Very good*	*Good*	*Reasonable*	*Poor*	*Very poor*	*Total*
Habitat of living, n (%)						
Village	348 (29.0)	596 (49.7)	212 (17.7)	39 (3.3)	5 (0.4)	1200 (100.0)
City <25,000 citizens	116 (28.9)	193 (48.0)	84 (20.9)	8 (2.0)	1 (0.2)	402 (100.0)
City 25,000–50,000 citizens	167 (27.8)	312 (52.0)	102 (17.0)	17 (2.8)	2 (0.3)	600 (100.0)
City 50,000–100,000 citizens	155 (27.0)	279 (48.7)	116 (20.2)	22 (3.8)	1 (0.2)	573 (100.0)
City >100,000 citizens	290 (32.5)	406 (45.5)	163 (18.3)	33 (3.7)	1 (0.1)	893 (100.0)
Total	1076	1786	677	119	10	3668
Level of education, n (%)						
Low	142 (23.5)	297 (49.3)	133 (22.1)	29 (4.8)	2 (0.3)	603 (100.0)
Middle	434 (28.8)	721 (47.8)	294 (19.5)	53 (3.5)	5 (0.3)	1507 (100.0)
High	500 (32.1)	768 (49.3)	250 (16.0)	37 (2.4)	3 (0.2)	1558 (100.0)
Total	1076	1786	677	119	10	3668

## DISCUSSION

To our knowledge, this study is the first to demonstrate the associations between the different domains of QoL and FI, constipation, and their coexistence in both men and women. Initially, the association did not seem strong, but after correcting for coexisting symptoms, the strength of the associations increased (see Supplemental Table 1 at http://links.lww.com/DCR/C260). This observation applied more strongly to respondents with constipation. The association between the severity of fecal problems and QoL was compared using the magnitudes of these associations of the QoL domains, as represented by the unstandardized regression coefficients (Fig. 1). Specifically, when the severity of constipation or FI increased by 1 point, the QoL lowered with the quantity of the unstandardized regression coefficient for that particular QoL domain (Fig. 1; see Supplemental Table 1 at http://links.lww.com/DCR/C260). When considering the severity of constipation, the association with QoL was strongest for role limitations due to emotional problems. Here, the unstandardized regression coefficient was –2.4, which indicates that when the severity of constipation increases by 1 point, the QoL domain score for role limitations due to emotional problems lowers by 2.4 points.

When considering the severity of FI, the association with QoL was strongest for role limitations due to physical problems, which entails, for example, less time spent working. Therefore, to the current understanding that the presence of fecal problems decreases QoL, we now add that the severity of fecal problems is associated with reduced QoL across all its domains in the study group, with constipation having stronger associations than FI.^3,5^

The general observation was that respondents rated their defecation health poorer as the severity of their fecal problems worsened. Nevertheless, a large group rated their defecation health as reasonable, good, or very good, although their severity scores were the highest (Fig. 4). This seems to indicate that respondents did not recognize their fecal problems as problematic, which withheld them from seeking health care support. This observation also emphasizes the need for physicians to actively heighten patient awareness regarding defecation health and what healthy defecation habits really look like. An alternative explanation for why patients may not be aware of their fecal problems despite having a reduced QoL is that the 2 problems might be unrelated.

To emphasize the importance of active and accurate screening for fecal problems, we analyzed the duration of bowel complaints and the severity of constipation and FI (Fig. 3). The results imply a relationship between long-standing bowel complaints and higher severity scores for constipation because respondents with lower severity scores reported a shorter duration of bowel complaints (Fig. 3). This indicates the need for timely treatment, even if the severity of the fecal problem is not yet severe. If not treated on time, bowel complaints will persist and will become more severe, as we observed that higher constipation severity scores existed in individuals who had bowel complaints for more than 20 years (Fig. 3). There seems to be a less clear-cut relationship between the duration of bowel complaints and FI severity. Nevertheless, we know that chronic constipation can progress to FI.^17^

In patients with FI, one study reported a slightly significant association between the severity of FI and QoL,^6^ whereas another study found no such association.^7^ In patients with chronic constipation, a slightly significant association was found between severity and QoL.^6^ We found significant associations between the severity of constipation and FI and every QoL domain (see Supplemental Table 1 at http://links.lww.com/DCR/C260). This could be partly explained by the fact that our study population consisted solely of respondents without bowel-related comorbidities. In the studies mentioned, the study populations consisted of patients with bowel-related comorbidities other than constipation and FI that can influence QoL. Furthermore, the fact that those patients were aware that they had fecal problems could also explain the difference between their results and ours. In our study, the respondents could be unaware that they had constipation, FI, or coexisting constipation and FI.

### Strength and Limitations

Previous studies involved patient populations, mostly in tertiary centers. By contrast, the primary strength of our study was that we studied the general population without bowel function comorbidities, which has not been done before. Thus, we prevented bowel-related comorbidities from influencing QoL. Previously, researchers pointed out that a more extensive study population would be needed to establish a more reliable association between the severity of fecal problems and QoL. A second strength of our study is our large study population of 3668 respondents. Third, the DeFeC and SF-36 questionnaires were completed digitally, so there were no missing data. Moreover, because the questionnaires were presented online and the data were studied anonymously, the answers may have been more reliable because it is perhaps less embarrassing to disclose fecal problems digitally. Another strength of this study was that apart from the DeFeC questionnaire, the respondents were also asked to complete the QoL questionnaire. This may have prevented bias in comparison to completing a questionnaire that focuses on health problems and includes questions on QoL.

This study is limited by its cross-sectional design and the fact that although many factors influence QoL, we only considered the severity of fecal problems. We chose not to correct our associations for age and BMI. A study performed by Silveira et al^18^ showed that among adults with obesity class II and III, 24.67% had constipation. This is comparable to the prevalence of 24.5% found in the study performed by Meinds et al.^1^ Furthermore, a study performed by Staller et al^19^ showed that there is no association between BMI and risk of FI. Because of the 2 aforementioned examples, we chose not to correct BMI. We decided not to correct for age because the proportion of the different age categories was comparable among each of the 4 groups (constipation, FI, both, neither).

Furthermore, the questionnaire was completed digitally, and therefore, there could be a selection bias toward healthy older adult respondents. Moreover, we did not ask about the economic status of the respondents, and this factor may contribute to the QoL. However, this bias is mildened by information about the educational level and habitat of living, which indirectly reflects on socioeconomic status and lifestyle. However, completing the questionnaires digitally also had many benefits, seeing that there were no missing data and we could include a large number of respondents in our study.

### Future Research and Recommendations

It would be interesting to compare the association between the severity of fecal problems and QoL in a healthy population without comorbidities and in a population with bowel comorbidities. It could be that coping behavior is different in these groups or that people with bowel comorbidities receive adequate therapy because of regular health care visits. Furthermore, it would be interesting to validate these results on a population from different countries and even more interesting, from other continents with different cultural backgrounds.

## CONCLUSIONS

The association between the severity of fecal problems and quality of life has not been investigated in the general population without bowel function comorbidities. The most striking result of our study is that the severity of constipation and FI is significantly negatively associated with QoL in a study population without bowel function morbidities. In general, in individuals having constipation, the association between severity and QoL is stronger than between FI severity and QoL. Interestingly, all our subgroups described their defecation health as very good, good, or reasonable. This could mean that fecal problems were not recognized because individuals had no idea what constituted normal defecation or the reluctance to disclose fecal problems was in play. Although constipation and FI are often not considered serious diseases, our study shows that even in respondents without other bowel-influencing comorbidities, QoL is significantly reduced with increasing severity of constipation and FI. Fecal problems do not resolve spontaneously and require treatment. Therefore, we advocate launching a prevention program for the general Dutch population to improve awareness concerning constipation and FI. Physicians should also inquire about patients’ defecation habits more actively and more adequately because patients do not voluntarily elaborate on this topic. In so doing, the more severe forms of fecal problems, which are more difficult to treat, could be prevented and unnecessary reduction of QoL could be avoided.

## ACKNOWLEDGMENTS

The authors thank T. van Wulfften Palthe, Ph.D., for correcting the English article.

## Supplementary Material
