# Supplementary material for: The Severity of Fecal Problems Is Negatively Associated With Quality of Life in a Dutch Population Without Bowel Function Comorbidities
Source: Dis Colon Rectum. 2023 Nov 2;67(3):448–56. doi: 10.1097/DCR.0000000000003048 (PMC10846588; doi:10.1097/DCR.0000000000003048)
Supplement: Supplementary file 2 [file dcr-67-448-s002.pdf]

Supplementary Table 1. The univariable and multivariable analysis of the severity of constipation and QoL and the severity of FI and QoL.

Associations between QoL and fecal problems	**Constipation**				**Fecal Incontinence**			
	**Univariable**		**Multivariable₁**		**Univariable**		**Multivariable₂**	
**QoL domains**	**Correlation coefficient (Pearson)**	***P* value**	**Unstandardized regression coefficient B (95% CI)**	***P* value**	**Correlation coefficient (Pearson)**	***P* value**	**Unstandardized regression coefficient B (95% CI)**	***P* value**
**Physical functioning**	-.188 *Very low**	0.000	-1.021 (-1.227 to -.815)	<.001	-.170 *Very low*	0.000	-1.032 (-1.337 to -.727)	<.001
**Social functioning**	-.328 *Low*	0.000	-2.201 (-2.430 to -1.972)	<.001	-.177 *Very low*	0.000	-0.871 (-1.210 to -.532)	<.001
**Role limitations (physical)**	-.259 *Low*	0.000	-1.955 (-2.226 to -1.685)	<.001	-.182 *Very low*	0.000	-1.280 (-1.681 to -.880)	<.001
**Role limitations (emotional)**	-.310 *Low*	0.000	-2.413 (-2.681 to -2.145)	<.001	-.171 *Very low*	0.000	-1.019 (-1.416 to -.621)	<.001
**Mental health**	-.332 *Low*	0.000	-1.864 (-2.053 to -1.675)	<.001	-.157 *Very low*	0.000	-0.568 (-.847 to -.288)	<.001
**Vitality**	-.330 *Low*	0.000	-1.920 (-2.115 to -1.725)	<.001	-.162 *Very low*	0.000	-0.599 (-.887 to -.310)	<.001
**Pain**	-.288 *Low*	0.000	-1.824 (-2.042 to -1.607)	<.001	-.164 *Very low*	0.000	-0.757 (-1.079 to -.435)	<.001
**General health perception**	-.257 *Low*	0.000	-1.420 (-1.616 to -1.224)	<.001	-.167 *Very low*	0.000	-0.816 (-1.106 to -.525)	<.001

1. Corrected for the severity of fecal incontinence, level of education and place of residence.
2. Corrected for the severity of constipation, level of education and place of residence.
